# Awareness of Risk Factors for Coronary Artery Disease in the Population of Al-Majma’ah Region, Saudi Arabia

**DOI:** 10.7759/cureus.52497

**Published:** 2024-01-18

**Authors:** Ahmed H Almutairi, Sultan A Alhassan, Faisal A Almutairi, Bader A Alharthi, Sulaiman A Aljabr, Riyadh A Alabdulmonem, Waleed M Altariqi, Ziad M Alaboody, Adel K Almutairy, Majed A AlQahtani, Osama M Albejede, Saud A Alrajeh, Mohammed D Alshahrani

**Affiliations:** 1 Emergency Medicine, Majmaah University, Al-Majma’ah, SAU; 2 College of Medicine, Majmaah University, Al-Majma’ah, SAU

**Keywords:** saudi arabia, al-majma'ah, awareness, cad, coronary artery disease

## Abstract

Background: Coronary artery disease (CAD) is a significant global health concern and a leading cause of morbidity and mortality. As a complex cardiovascular condition, CAD arises from the accumulation of atherosclerotic plaques within the coronary arteries, leading to restricted blood flow to the heart muscle. While CAD has been extensively studied, its prevalence remains a challenge, particularly in diverse populations with distinct cultural and lifestyle practices.

Objectives: To assess the awareness of risk factors for CAD in the population of Al-Majma'ah Region, Saudi Arabia.

Methods: The purpose of this cross-sectional descriptive study was to determine participants' awareness of CAD risk factors among the population of Al-Majma'ah Region, Saudi Arabia. It was conducted by the use of a self-administered questionnaire that had been validated in prior research publications. Sociodemographic information as well as the prevalence of cardiovascular disease risk factors were covered in the survey. The data analysis was done using IBM SPSS Statistics for Windows, Version 26 (Released 2019; IBM Corp., Armonk, New York, United States).

Results: A total of 919 individuals were enrolled in the current study after meeting the inclusion criteria. The results showed that most of the respondents 626 (68.1%) had a good level of awareness, 261 (28.4%) had a fair level of awareness, while only 32 (3.5%) of the respondents had a poor level of CAD risk factors awareness.

Conclusion: The majority of participants had a good level of knowledge regarding CAD risk factors. The correlation between monthly income and awareness of coronary artery risk factors was statistically significant.

## Introduction

Cardiovascular diseases (CVDs) are defined by the World Health Organization as the major cause of death around the world with an estimated number of 17.5 million of these cases happening in 2017, and 42.3% of these deaths were accounted to coronary artery disease (CAD) [1]. CAD is one of the most common cardiovascular disorders responsible for an increased disability as well as a decreased quality of life globally [2].

The prevalence of CAD is elevated in nations that are both industrialized and developing. Based on one study, 32.7% of CVDs and 2.2% of the global disease burden are believed to be a result of CAD. It results in an annual cost to the US healthcare system of over 200 billion dollars. The American Heart Association's (AHA) national health survey from 2009 to 2012 estimates that 5.0% of women and 7.6% of men in the US had CAD. This translates to 15.5 million Americans who had the illness at this time [3,4].

CAD has a wide spectrum from a human who is asymptomatic to another presenting with acute coronary syndromes (ACSs) or presenting dead. Coronary atherosclerotic disease is a long-term, advancing process that leads to atherosclerotic plaque advancement and progression within the heart's coronary arteries [5].

The illness known as CAD is characterized by an insufficient flow of blood and oxygen to the heart muscle. Oxygen demand and supply are out of balance because of coronary artery blockage. Usually, it is caused by plaques that obstruct blood flow in the coronary artery lumen. In the US and around the world, it is the leading cause of death. It was an unusual cause of death during the start of the 20th century. Though the number of deaths from CAD declined after reaching a peak in the middle of the 1960s, it remains the biggest cause of death globally [6].

The phenomenon of CAD is multifactorial. There are non-modifiable and modifiable factors which are the two main categories of risk factors. Genetics, age, family history, and gender are examples of non-modifiable factors. Risk factors that can be modified include lipid levels, obesity, smoking, and psychosocial factors. Because of a faster-paced lifestyle and greater consumption of fast food and unhealthy meals, CAD is becoming more common in the Western world. In the US, the incidence has become more common towards a later age due to improved primary care in the middle and upper socioeconomic classes. The leading cause of cardiovascular illnesses is still smoking. The adult smoking prevalence in the United States was determined to be 15.5% in 2016 [7].

Compared to the feminine gender, the male gender is more predisposed. Unchangeably, hypercholesterolemia is still a significant risk factor for CAD. Elevated high-density lipoproteins (HDLs) reduce the incidence of CAD, while increased low-density lipoproteins increase the risk for CAD. Atherosclerotic cardiovascular disease (ASCVD) 10-year risk can be computed online at the American Heart Association portal using the ASCVD equation. Inflammation markers are also potent coronary heart disease risk factors. According to certain research, high-sensitivity CRP is the most accurate indicator of CAD, yet its practical applications are debatable [8].

The main complications associated with CAD are cardiac arrhythmia ACS, congestive cardiac failure, regurgitation of the mitral valve, ventricular free wall rupture, pericarditis, aneurysm formation, and mural thrombi [9-11].

The lack of awareness often hinders timely risk factor modification, early symptom recognition, and appropriate health-seeking behaviors, leading to preventable adverse cardiovascular events. Therefore, efforts to enhance public awareness and knowledge about CAD and its risk factors are imperative for reducing the disease burden and improving population health outcomes.

This research aims to assess the current level of awareness and understanding of CAD and its risk factors among the population of Al-Majma'ah Region, Saudi Arabia.

## Materials and methods

Study design and setting

A descriptive cross-sectional study was conducted. A pilot study was performed for the questionnaire, focusing on the initial validation and refinement through a small-scale survey. The questionnaire, developed based on a comprehensive literature review related to CAD awareness [12], was assessed by a total of 20 participants to evaluate the clarity, comprehensibility, and relevance of the items. Following the pilot survey, preliminary data were analyzed to identify potential issues and areas for improvement. The findings from this pilot study contributed to finalizing the questionnaire for use in a larger-scale study assessing CAD awareness in the target population, conducted in Al-Majma'ah Region, Saudi Arabia.

Target population, sampling, and duration of study

The population in the Al-Majma'ah Region. The sample size is calculated using the formula n=(z^2 x pq)/d^2, where n represents the sample size, z is the standard deviation (1.96), p is the prevalence (0.5), q is 1-p, DE is the design effect (2), and d is the accepted error (0.05), was determined to be 384. Participants were selected through non-probability convenience sampling. The study lasted for six months.

Data collection

Data were collected using a pre-tested and self-administered questionnaire. IBM SPSS Statistics for Windows, Version 26 (Released 2019; IBM Corp., Armonk, New York, United States) was employed for data analysis. The questionnaire included information on age, gender, and awareness questions on CAD.

Inclusion and exclusion criteria

The study included Saudi and non-Saudi nationals aged ≥18 years, mentally competent, and residing in the Al-Majma'ah Region. Exclusions were made for those below 18 years of age.

Data analysis

IBM SPSS Statistics for Windows, Version 26 (Released 2019; IBM Corp., Armonk, New York, United States) was used for statistical analysis. Descriptions of categorical variables utilized frequency and percentiles, while ongoing variables were described using mean and standard deviations. The Chi-squared test, t-test, and analysis of variance (ANOVA) were employed to evaluate differences and relationships between variables. The significance threshold (p-value) was set at 0.05 with a 95% confidence interval.

Ethical consideration

Ethics approval was obtained from Majmaah University for Research Ethics Committee (MUREC-Dec.25 / COM-2023/ 36-6). Informed consent was obtained from participants, and all collected data were kept confidential and used solely for the purpose of this study.

## Results

Table 1 shows the sociodemographic information of the participants. About 381 (41.5%) of the 919 participants were male, and 538 (58.5%) were female. Further, most of the participants, 689 (75.0%), were between the ages of 18 and 25, 141 (15.3%) were between the ages of 26 and 35, 53 (5.8%) were between 36 and 45, and 36 (3.9%) were over 45. In addition, most of the participants, 745 (81.1%), were single, followed by married individuals, 144 (15.7%), divorced individuals, 180 (19.1%), and widowed individuals, 11 (1.2%). When it came to monthly income, 242 participants (26.3%) indicated that they made less than 5000 Saudi Riyal (SAR), 224 participants (24.4%) made between 5000 and 9999 SAR, 180 participants (19.6%) made between 10000 and 14999 SAR, and 273 participants (29.7%) indicated that they made more than 15000 SAR.

**Table 1:** Socio-demographic characteristics of the participants (n=919) Data has been presented as n, %. SAR: Saudi Riyal

Variables	Characteristics	N (%)
Gender	Male	381 (41.5%)
	Female	538 (58.5%)
Age	18-25 years	689 (75.0%)
	26-35 years	141 (15.3%)
	36-45 years	53 (5.8%)
	Over 45 years	36 (3.9%)
Marital status	Single	745 (81.1%)
	Married	144 (15.7%)
	Divorced	19 (2.1%)
	Widowed	11 (1.2%)
Monthly income	Less than 5000 SAR	242 (26.3%)
	5000-9999 SAR	224 (24.4%)
	10000-14999 SAR	180 (19.6%)
	Over 15000 SAR	273 (29.7%)

Table 2 shows the respondents' knowledge of the different risk factors linked to coronary heart disease. About 868 (94.5%) of the respondents were aware that CVD is more common in smokers. In a similar vein, 718 (78.1%) of participants thought that walking for at least 30 minutes a day for five days raises the risk of CVD. Furthermore, 794 (86.4%) of participants acknowledged that consuming fast food elevates the likelihood of developing CVD. Approximately 734 (79.9%) of participants thought that drinking soft drinks raises the risk of heart disease. Of the respondents, 553 (60.2%) knew that CVD and aging are related. Moreover, 515 (56.3%) of participants were aware that an individual's risk is elevated if they have a family member with CVD. When it comes to particular factors, 805 (87.6%) of respondents acknowledged that having high blood cholesterol raises the risk of CVD. In a similar vein, 712 (77.5%) of participants knew that diabetes, or elevated blood sugar, raises the risk. According to 824 (89.7%) of respondents, obesity raises the risk of CVD. Moreover, 752 (81.8%) of participants acknowledged that stress and anxiety raise the risk of heart disease. Regarding gender, 501 (54.5%) of participants thought that men were more prone to heart disease than women. Finally, 789 (85.9%) of participants were aware that high blood pressure raises the chance of developing CVD.

**Table 2:** Awareness of coronary disease risk factors (n=919) Data has been presented as n, %.

Variables	Responses	N (%)
Smokers are more likely to have cardiovascular disease	Yes	868 (94.5%)
	No	51 (5.5%)
Exercising at least 30 minutes of walking daily for five days increases the incidence of cardiovascular disease	Yes	718 (78.1%)
	No	201 (21.9%)
Eating fast food increases the risk of cardiovascular disease	Yes	794 (86.4%)
	No	125 (13.6%)
Soft drinks increase the risk of cardiovascular disease	Yes	734 (79.9%)
	No	185 (20.1%)
Age is linked to cardiovascular disease	Yes	553 (60.2%)
	No	366 (39.8%)
Having a family member with cardiovascular disease increases your risk of cardiovascular disease	Yes	517 (56.3%)
	No	402 (43.7%)
High cholesterol in the blood increases the risk of cardiovascular disease	Yes	805 (87.6%)
	No	114 (12.4%)
High blood sugar (diabetes) increases the risk of cardiovascular disease	Yes	712 (77.5%)
	No	207 (25.5%)
Obesity increases the risk of cardiovascular disease	Yes	824 (89.7%)
	No	95 (10.3%)
Anxiety and stress increase the risk of cardiovascular disease	Yes	752 (81.8%)
	No	167 (18.2%)
Males are more susceptible to cardiovascular disease than female	Yes	501 (54.5%)
	No	418 (45.5%)
High blood pressure increases the risk of cardiovascular disease	Yes	789 (85.9%)
	No	130 (14.1%)

Table 3 demonstrates the awareness score ranged from 0 to 12 whereby the scores in the range of 0-4 indicated poor, 5-8 indicated fair and 9-12 indicated good. The average awareness score was 9.32±2.45. As shown in Table 3, most of the respondents 626 (68.1%) demonstrated a good level of awareness 261 (28.4%) of the respondents had a fair level of awareness, while only 32 (3.5%) of the respondents had a poor level of CAD awareness.

**Table 3:** Level of awareness about risk factors for coronary artery disease Data has been presented as n, %.

Scores	Frequency (n)	Percentage (%)
Poor	32	3.5%
Fair	261	28.4%
Good	626	68.1%

Table 4 shows the relationship between the participants' awareness of coronary artery risk factors and their sociodemographic traits. Based on the results, a statistically significant correlation between monthly income and awareness of coronary artery risk factors is indicated by the p-value of 0.001.

**Table 4:** Association between coronary artery risk factors awareness and socio-demographic characteristics (n=919) Data has been presented as n, %. *A p-value less than 0.05 was considered statistically significant. SAR: Saudi Riyal

Variables	Category	Poor N (%)	Fair N (%)	Good N (%)	p-values*
Gender	Male	19 (5.5%)	99 (26.0%)	263 (69.0%)	0.059
	Female	13 (2.4%)	162 (30.1%)	363 (67.5%)	
Age	18-25 years	26 (3.8%)	200 (29.0%)	463 (67.2%)	0.394
	26-35 years	4 (2.8%)	43 (30.5%)	141 (66.7%)	
	36-45 years	1 (1.9%)	8 (15.1%)	44 (83.0%)	
	Over 45 years	1 (2.8%)	10 (27.8%)	25 (69.4%)	
Marital status	Single	30 (4.0%)	216 (29.0%)	499 (67.0%)	0.475
	Married	2 (1.4%)	37 (25.7%)	105 (72.9%)	
	Divorced	0 (0.0%)	4 (21.1%)	15 (78.9%)	
	Widowed	0 (0.0%)	4 (36.4%)	7 (63.6%)	
Monthly income	Less than 5000 SAR	4 (1.7%)	77 (31.8%)	161 (66.5%)	0.001
	5000-9999 SAR	2 (0.9%)	61 (27.2%)	161 (71.9%)	
	10000-14999 SAR	3 (1.7%)	43 (23.9%)	134 (74.4%)	
	Over 15000 SAR	23 (8.4%)	80 (29.3%)	170 (62.3%)	

## Discussion

The purpose of this study was to assess the current level of awareness and understanding of CAD and its risk factors among the population of Al-Majma'ah Region, Saudi Arabia. As revealed in the study findings 868 (94.5%) of the respondents were aware that CVD is more common in smokers. Similarly, 718 (78.1%) of the participants indicated that walking for at least 30 minutes a day for five days raises the risk of CVD. In addition, most of the participants 794 (86.4%) acknowledged that consuming fast food elevates the likelihood of developing CVD. This was consistence with the findings obtained in a study by Hajar which noted that the most widespread knowledge of CAD risk factors was smoking. The frequency of knowledge about risk factors was 92%, 81%, 79%, 55%, 48%, and 39%, respectively. These risk factors included smoking, fatty food, obesity, hypertension, high cholesterol, and diabetes mellitus [13].

Further, the study revealed that most 734 (79.9%) of the participants understood that drinking soft drinks raises the risk of heart disease and 553 (60.2%) knew that CVD and aging are related. Moreover, 515 (56.3%) of participants were aware that an individual's risk is elevated if they have a family member with CVD. When it comes to particular factors, 805 (87.6%) of respondents acknowledged that having high blood cholesterol raises the risk of CVD. In relation to a study by Li et al., hypertension, diabetes, and dyslipidemia awareness had prevalence rates of 56.6%, 28.3%, and 25.1%, and 91.3%, 40.9%, and 92.0%, respectively. The study noted a significant difference in the prevalence of cardiometabolic risk factors based on both disease status and gender [14]. Further, Rasheed et al. in their study revealed the prevalence of CAD risk factors awareness as diabetes (59.3%), hypertension (80.7%), dietary fat (79%), stress (75.5%), smoking (77.5%), sedentary lifestyle (68%), obesity (66%), advanced age (65.2%), male gender (52.7%), and family history (52%) [15].

Moreover, the study findings reveal that 712 (77.5%) of the participants knew diabetes, or elevated blood sugar, raises the risk. Further, it noted that 824 (89.7%) of the respondents understood that obesity raises the risk of CVD, 752 (81.8%) acknowledged that stress and anxiety raise the risk of heart disease, 501 (54.5%) knew that men were more prone to heart disease than women while 789 (85.9%) of the participants were aware that a high blood pressure raises the chance of developing CVD. In line with a study by Sekhri et al., high total cholesterol/HDL ratio, dyslipidemia was significantly more common among patients with CAD [16].

The study also found that the average CAD awareness score was 9.32±2.45 with most of the respondents 626 (68.1%) showing a good level of CAD awareness while 261 (28.4%) of the respondents had a fair level of awareness. The study revealed a statistically significant correlation between monthly income and awareness of coronary artery risk factors (p=0.001). A study by Matysek et al., also noted that the CAD risk factors knowledge score was higher. 20 (12-24) vs. 22 (19-25) (points, per 31 max.) were the median (IQR) results in the prior-PCI group compared to the prior-CABG group, respectively; p=0.01. The degree of risk control showed similar outcomes (prior-PCI vs. prior-CABG, respectively: 6 (4-7) vs. 7 (6-8) (points, per 15 max.); (p=0.002) [17]. The average score for general awareness was 4.31±1.36 (1.00-8.00). As noted in another study, the most often mentioned risk factors were soft drinks, fast food, and a family history of diabetes, which were reported by 74.8%, 64.3%, and 47.2% of participants, respectively [18]. The total awareness score and the knowledge of each risk factor separately showed a significant correlation (p<0.003) [19].

The study's limitations are that the online questionnaire used to gather data for the study could have an impact on its validity if the responses were examined. Further, the population of Saudi Arabia's Al-Majma'ah Region is insufficient to adequately represent the country's total population, so extrapolating the findings to the entire country is not possible.

## Conclusions

The majority of participants had good knowledge of CADs. There are some information gaps about age, gender, and marital status, among other factors. Household monthly income was associated with higher CAD risk factors knowledge levels. Programs for health education and increased media involvement in information dissemination should be used to fill the knowledge gaps.
